# Clinical results of multidisciplinary therapy including palliative posterior spinal stabilization surgery and postoperative adjuvant therapy for metastatic spinal tumor

**DOI:** 10.1186/s13018-018-0735-z

**Published:** 2018-02-05

**Authors:** Hiroshi Uei, Yasuaki Tokuhashi, Masafumi Maseda, Masahiro Nakahashi, Hirokatsu Sawada, Enshi Nakayama, Hirotoki Soma

**Affiliations:** Department of Orthopaedic Surgery, Nihon University School of Medicine, 30-1 Oyaguchi Kami-cho, Itabashi-ku, Tokyo, 173-8610 Japan

**Keywords:** Spinal metastases, Palliative surgery, Tokuhashi score, Minimally invasive spine stabilization, Multidisciplinary therapy

## Abstract

**Background:**

Surgeries performed for metastatic spinal tumor are mostly palliative and are controversial for patients with short life expectancy. We investigated whether palliative posterior spinal stabilization surgery with postoperative multidisciplinary therapy results in improvement of life prognosis and activities of daily living (ADL) in patients with metastatic spinal tumor.

**Methods:**

The subjects were 55 patients who underwent palliative posterior-only instrumentation surgery for metastatic spinal tumor at our hospital between 2012 and 2015. Postoperative survival, early paralysis improvement, ADL improvement, and rate of discharge to home were examined.

**Results:**

The patients included 37 males and 18 females, and the mean age at the time of surgery was 66.8 years old. The mean Tokuhashi score was 7.1, the mean spinal instability neoplastic score (SINS) was 9.4, and the epidural spinal cord compression scale (ESCCS) was grade 3 in 20 patients (36.3%). The mean Barthel index for ADL was 48.7. The median postoperative survival time determined using the Kaplan-Meier method was 12.0 months (95% confidence interval 2.4–21.5). Regarding improvement of paralysis, the modified Frankel scale was improved by one grade or more or grade E was maintained in 35 patients (63.6%), whereas paralysis aggravated in 2 (3.6%). In surgery, conventional posterior decompression and fixation were applied in 31 patients (56.3%), and minimally invasive spine stabilization was applied in 24 (43.6%). Postoperative chemotherapy was performed in 31 patients (56.3%), radiotherapy was used in 38 (69.0%), and a bone-modifying agent was administered in 39 (70.2%). Regarding ADL, the mean Barthel index improved from 48.5 before surgery to 74.5 after surgery. Thirty-seven patients (67.2%) were discharged to home.

**Conclusions:**

ADL improved and allowed discharge to home, and postoperative adjuvant therapy could be administered at a high rate in patients who received palliative posterior spinal stabilization surgery. Survival time extended beyond the preoperative life expectancy in many patients. Patients with a metastatic spinal tumor have short life expectancy and paralysis caused by spinal instability and spinal cord compression. However, multidisciplinary therapy including palliative posterior spinal stabilization surgery with reduced invasiveness and postoperative adjuvant therapy are effective in these patients.

## Background

Most cases of metastatic spinal tumor are systemic conditions with few treatment options [1–3]. Cases of symptomatic spinal metastasis have increased because the survival time of patients with metastatic cancer has been extended by advances in cancer therapy [4]. Metastatic spinal tumor destroys the spine, which induces collapse of spinal support, infiltration into the spinal cord and cauda equina, and compression, which cause pain, paralysis, and disruption of activities of daily living (ADL). Surgeries performed for metastatic spinal tumor are mostly palliative, and use of these procedures in patients with short life expectancy is controversial [5].

Surgical treatment for metastatic spinal tumor with reduced invasiveness is now available, including minimally invasive spine stabilization (MISt) with percutaneous pedicle screws (PPSs) [6–8] and balloon kyphoplasty [9]. Cancer chemotherapy has also advanced [10], and bone-modifying agents [11] for bone metastasis have been introduced. However, only a few studies have investigated whether these new treatments for metastatic spinal tumor contribute to life expectancy and ADL improvement. In this study, we examined use of not only conventional posterior decompression and fixation surgery but also MISt as palliative surgery and the frequency of postoperative adjuvant therapy for metastatic spinal tumor, with a focus on how much MISt contributed to decreasing surgical stress, early paralysis improvement, extended survival, ADL improvement, and rate of discharge to home.

## Methods

### Patient population

After institutional review board approval was obtained, we reviewed our institutional database for patients who had undergone surgery for metastatic spinal tumors. The subjects were 55 patients who underwent palliative surgery for metastatic spinal tumor at our hospital between 2012 and 2015. Inclusion criteria were patients planned for palliative surgery for metastatic spinal tumors that were treated with posterior-only instrumentation. Patients were excluded if they had concurrent anterior fusion surgery. Demographic data, presenting symptoms, and radiographic studies were reviewed.

### Surgical indication

**Fig. 1:**
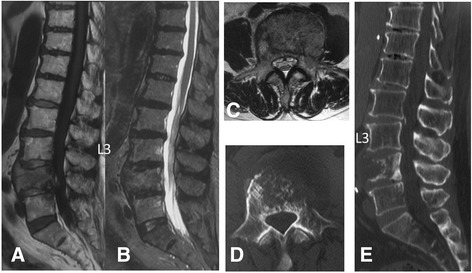
A 64-year-old man with metastasis of liver cancer to the 4th and 5th lumbar vertebrae. The Tokuhashi score was 3. Minimally invasive spine stabilization (MISt) without decompression (L2-S2AI) was applied. The operative time was 182 min, and blood loss was 152 ml. The grade of paralysis improved from D1 before surgery to E after surgery. **a** Sagittal view on preoperative T1-weighted MRI. **b** Sagittal view on preoperative STIR MRI. **c** Axial view at L4 on preoperative T2-weighted MRI. **d** Axial view at L4 on preoperative plain CT. **e** Sagittal view on preoperative plain CT

**Fig. 2:**
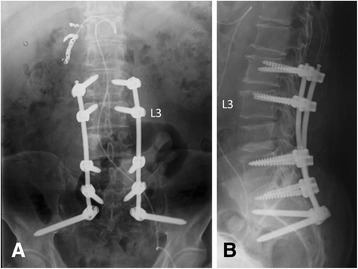
Postoperative day 3. **a** Posteroanterior view on postoperative radiography. **b** Lateral view on postoperative radiography

The indications for palliative surgery for metastatic spinal tumor [1, 2] are (1) intractable pain due to spinal instability or threat of instability defined by SINS [12], (2) spinal paralysis such as any change in the motor examination, and (3) radiation-resistant cancer such as kidney cancer or thyroid cancer. The exclusion criteria are (1) case indicated for total en bloc spondylectomy, (2) life expectancy < 6 months and responsive to narcotic analgesics or markedly responsive to radiotherapy, and (3) poor general condition (Karnofsky performance status ≥ 3) and reduced will to live. Patients were treated primarily with MISt as much treatment as possible was provided (Figs. 1 and 2). The exclusion criteria for not treatable with MISt were (1) lesions in the occipital over the cervical region and (2) difficulty confirming the pedicle of the vertebral arch under a fluoroscope or in PPS insertion. When impossible to treat with MISt, patients were treated with conventional posterior decompression and fixation surgery.

### Outcome evaluation

The evaluation items were (1) postoperative survival time; (2) paralysis improvement; (3) surgical procedure, including number of levels fused, operative time, intraoperative blood loss, and complications; (4) use of postoperative adjuvant therapy with chemotherapy, radiotherapy, and bone-modifying agents; (5) change in Barthel index for ADL from before surgery to the highest value after surgery; (6) rate of discharge to home; and (7) VAS at 2 weeks after surgery. With respect to postoperative adjuvant therapy, we tried to perform radiotherapy after surgery excluding radiation-resistant cancer, as long as it is not inconvenient for treatment of the primary cancer. The criterion for discharge to home was Barthel index ≥ 70 or availability of sufficient care by family members for cases with Barthel index < 70. Statistical analysis was performed using SPSS v.19.0 (SPSS Inc., Chicago, IL, USA), with the significance level at 5%.

## Results

The patients included 37 males (67.2%) and 18 females (32.7%), and the mean age at the time of surgery was 66.8 years old (Table 1). The primary lesion was lung cancer in 11 patients (20.0%), liver cancer in 9 (16.3%), prostate cancer in 6 (10.9%), myeloma in 5 (9.0%), kidney in 4 (7.2%), thyroid in 4 (7.2%), lymphoma in 3 (5.4%), gallbladder in 3 (5.4%), breast in 2 (3.6%), sarcoma in 2 (3.6%), others in 5 (9.0%), and unknown in 1 (1.8%). The level of the main lesion was the cervical spine in 9 patients (16.3%), thoracic spine in 34 (58.1%), and lumbar spine in 14 (25.4%). The grade of preoperative paralysis on the modified Frankel scale (Table 2) [13] was A in 2 patients (3.6%), B in 1 (1.8%), C in 19 (34.5%), D1 in 15 (27.2%), D2 in 1 (1.8%), D3 in 10 (18.1%), and E in 7 (12.7%). The mean preoperative visual analogue scale (VAS) for pain was 4.6. The Tokuhashi score [1–3] was 0–8, indicating life expectancy < 6 months, in 40 patients (72.7%); 9–11, life expectancy ≥ 6 months, in 9 (16.3%); and 12–15, life expectancy ≥ 1 year, in 6 (10.9%); with an overall mean of 7.1. The spinal instability neoplastic score (SINS) [12] was 0–6, indicating instability, in 6 patients (10.9%); 7–12, threat of instability, in 48 (87.2%); and 13–18, stability, in one (1.8%); with a mean of 9.4. The epidural spinal cord compression scale (ESCCS) [14] for nerve compression was grade 0, indicating a tumor restricted to the bone, in one patient (1.8%); grade 1a, infiltration in the spinal canal, in 4 (7.2%); grade 1b in 4 (7.2%); grade 1c in 3 (5.4%); grade 2, nerve compression, in 23 (41.8%); and grade 3, marked exclusion of the nerve, in 20 (36.3%). The mean preoperative Barthel index [15] for ADL was 48.7.

**Table 1:** Baseline characteristics

Characteristic	Value
Patients, *n*	55
Age at surgery, mean (range), years	66.8 (26–92)
Sex, *n* (%)	
Male	37 (67.2)
Female	18 (32.7)
Metastatic tumor diagnosis, *n* (%)	
Lung	11 (20.0)
Liver	9 (16.3)
Prostate	6 (10.9)
Myeloma	5 (9.0)
Kidney	4 (7.2)
Thyroid	4 (7.2)
Lymphoma	3 (5.4)
Gallbladder	3 (5.4)
Breast	2 (3.6)
Sarcoma	2 (3.6)
Others	5 (9.0)
Unknown	1 (1.8)
Main level of tumors, *n* (%)	
Cervical	9 (16.3)
Thoracic	32 (58.1)
Lumbar	14 (25.4)
Preoperative modified Frankel category, *n* (%)	
A	2 (3.6)
B	1 (1.8)
C	19 (34.5)
D1	15 (27.2)
D2	1 (1.8)
D3	10 (18.1)
E	7 (12.7)
Visual analogue scale (range)	4.6 (1–10)
Tokuhashi score, *n* (%)	
0–8	40 (72.7)
9–11	9 (16.3)
12–15	6 (10.9)
Spinal instability neoplastic score, *n* (%)	
0–6	6 (10.9)
7–12	48 (87.2)
13–18	1 (1.8)
Epidural spinal cord compression scale, *n* (%)	
0	1 (1.8)
1a	4 (7.2)
1b	4 (7.2)
1c	3 (5.4)
2	23 (41.8)
3	20 (36.3)
Preoperative Barthel index, mean (range)	48.7 (0–100)

**Table 2:** Modified Frankel grading scale

Grade	Neurological status
A	Complete motor and sensory loss
B	Preserved sensation only, voluntary motor function absent
C	Preserved motor less than fair grade (nonfunctional for any useful purpose)
D1	Preserved motor at lowest functional grade (3 +/5 +) and/or with bowel or bladder dysfunction
D2	Preserved motor at midfunctional grade (3 + to 4 +/5 +) and/or neurologic bowel or bladder dysfunction
D3	Preserved motor at high-function grade (4 + to 5 +) and normal voluntary bowel or bladder function
E	Complete motor and sensory function normal (may still have abnormal reflexes)

**Fig. 3:**
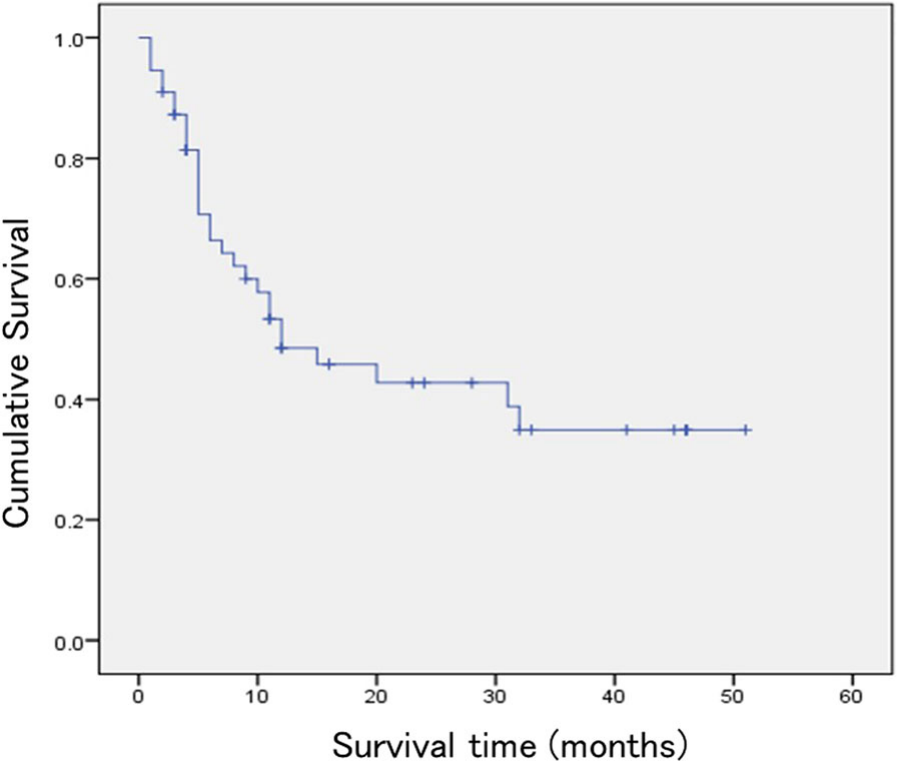
Kaplan-Meier curve for survival of patients after palliative surgery for metastatic spinal tumor. The median survival time was 12.0 months (95% confidence interval 2.2–21.8)

The median postoperative survival time determined using the Kaplan-Meier method was 12.0 months (95% confidence interval 2.4–21.5) (Fig. 3). As for two sarcoma patients, they died 10 and 15 months after surgery. Regarding five myeloma patients, they were all alive at 4 to 30 months (mean 11.6 months) after surgery. Paralysis was improved by ≥ 1 grade or grade E was maintained in 35 patients (63.6%) (Table 3). The grade was C or lower before surgery in 22 patients (40.0%), but the number of patients with these grades decreased to 12 (21.8%) after surgery. The postoperative grade was E in 21 patients (38.1%). Paralysis aggravated in 2 patients (3.6%).

**Table 3:** Neurological recovery on the modified Frankel scale

MFS	Number of cases before surgery	Number of cases after surgery						
		A	B	C	D1	D2	D3	E
A	2	2	0	0	0	0	0	0
B	1	1	0	0	0	0	0	0
C	19	0	1	7	3	1	1	6
D1	15	0	0	0	2	4	5	4
D2	1	0	0	0	0	1	0	0
D3	10	0	0	1	0	0	4	5
E	7	0	0	0	0	0	1	6
Total	55	3	1	8	5	6	11	21

The surgical procedure was conventional posterior decompression and fixation in 31 patients (56.3%) and MISt in 24 (43.6%) (Table 4). The mean operative time was 201 min, the mean intraoperative blood loss was 474 ml, and the mean number of levels fused was 5.5. Perioperative complications included massive bleeding of ≥ 1500 ml during surgery in 5 patients (9.0%), death within 30 days after surgery in 3 patients (5.4%), epidural hematoma after surgery in 2 (3.6%), and wound dehiscence in 2 (3.6%). With regard to two postoperative epidural hematoma patients, they were treated with surgical evacuation immediately after diagnosis. As for two patients with wound dehiscence, they were treated with resuture and healed of wound properly.

**Table 4:** Intraoperative parameters

Variable	Value
Procedures	
Conventional posterior decompression and fixation, *n* (%)	31 (56.3)
Minimally invasive spine stabilization, *n* (%)	24 (43.6)
Number of levels fused, mean (range)	5.5 (2–11)
Operation time, mean (range), (min)	201 (63–371)
Blood loss, mean (range), (ml)	474 (5–4140)
Perioperative complications, yes, *n* (%)	
Massive bleeding (> 1500 ml)	5 (9.0)
Early death (within 30 days postoperatively)	3 (5.4)
Epidural hemorrhage	2 (3.6)
Wound dehiscence	2 (3.6)
Upper airway obstruction	1 (1.8)
Acute renal failure	1 (1.8)
Surgical site infection	1 (1.8)
Deep vein thrombosis	1 (1.8)

**Fig. 4:**
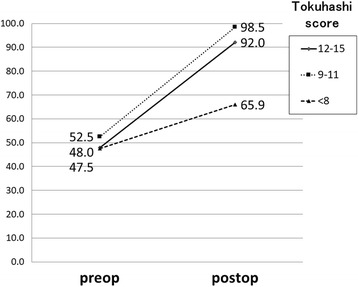
Changes in Barthel index after surgery in patients classified by Tokuhashi score for life expectancy before surgery. The mean Barthel index in all patients improved from 48.7 before surgery to 74.3 after surgery

As postoperative adjuvant therapy, chemotherapy was performed in 31 patients (56.3%), radiotherapy was used in 38 (69.0%), and a bone-modifying agent was administered in 39 (70.2%) (Table 5). The mean preoperative VAS for pain improved from 4.6 before surgery to 0.9 after surgery (*P* < 0.001), and the mean preoperative Barthel index for ADL improved from 48.5 before surgery to 74.5 after surgery (*P* < 0.001). In patients with mean preoperative Tokuhashi scores of ≤ 8, 9–11, and ≥ 12, the mean Barthel index improved from 47.5 to 65.9 (*P* < 0.001), 52.5 to 98.5 (*P* = 0.01), and 48.0 to 92.0 (*P* < 0.001), respectively (Fig. 4). Thirty-seven patients (67.2%) were discharged to home, including 48.8% (20/41), 66.6% (6/9), and 100% of patients with preoperative Tokuhashi scores of ≤ 8, 9–11, and ≥ 12, respectively.

**Table 5:** Outcomes after surgery

Variable	Value
Additional adjuvant therapy, *n* (%)	
Chemotherapy	31 (56.3)
Radiotherapy	38 (69.0)
Bone-modifying agent	39 (70.2)
Visual analogue scale (range)	0.9 (0–4)
Postoperative Barthel index, mean (range)	74.5 (0–100)
Postoperative course, *n* (%)	
Discharge to home	37 (67.2)
Transfer to hospice	11 (20.0)
In-hospital death	7 (12.7)

## Discussion

Health conditions and quality of life (QOL) may be improved in survivors of advanced cancer with sufficient survivorship care [16]. Prevention of spinal metastasis-induced skeletal-related events (SREs) is an important aspect of survivorship care because pathological fracture is associated with increased mortality [17]. Surgery alone is insufficient to strengthen survivorship care for patients with metastatic spinal tumor, and administration of adjuvant therapy including radiotherapy, chemotherapy appropriate for the cancer type, and a bone-modifying agent (bisphosphonate or denosumab) followed by hormone replacement are necessary. However, there has been a tendency to focus only on the surgical method and short-term improvement of paralysis in evaluation of therapeutic effects.

Given that improvement of outcomes by surgical treatment alone is limited, we used chemotherapy, radiotherapy, and bone-modifying agents after surgery in 56, 69, and 70% of cases in this study, respectively. Of the cases excluding radiation-resistant cancer such as kidney and thyroid cancer, radiotherapy was performed in 38 out of 47 patients (80%). This resulted in improved ADL and greater discharge to home, compared with findings in our previous study [1]. The rate of conventional posterior decompression and fixation surgery decreased to 56%, whereas MISt increased. Performance of MISt at an early stage may have improved ADL, allowed discharge to home, and facilitated use of postoperative adjuvant therapy.

Accurate preoperative prediction of the outcome of surgery for metastatic spinal tumor is difficult [1–3, 5], and this makes the significance of palliative surgery for patients with short life expectancy controversial. The mean Tokuhashi score was 7.1 in all patients and the score was 0–8 in 40 patients (72.7%), indicating life expectancy < 6 months. Performance of palliative surgery in patients with such a short life expectancy requires consideration of the risk of complications, expected benefits, and medical costs [18]. However, there is also the opinion that palliative surgery should be performed [19] because improvement of ADL by surgery may increase the opportunity for adjuvant therapy after surgery and indirectly extend survival. We also consider that survival can be extended beyond the preoperative life expectancy and ADL can be improved by multidisciplinary therapy including palliative posterior spinal stabilization surgery with reduced invasiveness and postoperative adjuvant therapy. Previous studies of minimally invasive posterior fixation as palliative surgery have focused on technical aspects, surgical invasiveness, and short-term improvement of paralysis, whereas few have examined outcomes of postoperative survival, ADL, and QOL. Improvement of QOL at 30 days after surgery [6] and median postoperative survival of 11.3 months [7] have been reported. In the current study, the median postoperative survival determined by the Kaplan-Meier method was 12.0 months, and the survival time was longer than the preoperative life expectancy in many patients. The mean Barthel index reflecting ADL improved to 74.5 after surgery, but this was not a marked improvement compared with that in our previous study [1]. In patients with mean preoperative Tokuhashi scores of ≤ 8, the mean Barthel index improved from 47.5 to 65.9. However, our criterion for discharge to home was Barthel index ≥ 70 or availability of sufficient care by family members for cases with Barthel index < 70.Hence, the rate of discharge to home was 67%, which showed a marked improvement but still not satisfactory.

Chemotherapy, radiotherapy, and treatment with bone-modifying agents can be performed at an outpatient clinic, and this may have contributed to improvement of postoperative patients’QOL. The limitations of this study were that the study design was retrospective without a control group, and there was insufficient evaluation of QOL, but the rate of patients treated with MISt increased, and MISt is advantageous in that the surgical wound is small and the wound heals rapidly. This may have facilitated early postoperative adjuvant therapy and led to the improved rate of discharge to home, which in turn resulted in improved QOL.

## Conclusions

ADL improved and allowed discharge to home, and postoperative adjuvant therapy could be administered at a high rate in patients who received palliative posterior spinal stabilization surgery. Survival time extended beyond the preoperative life expectancy in many patients. Patients with a metastatic spinal tumor have short life expectancy and paralysis caused by spinal instability and spinal cord compression. However, multidisciplinary therapy including palliative posterior spinal stabilization surgery with reduced invasiveness and postoperative adjuvant therapy are effective in these patients.

## Abbreviations

ADL: Activities of daily living
ESCCS: Epidural spinal cord compression scale
MISt: Minimally invasive spine stabilization
QOL: Quality of life
SINS: Spinal instability neoplastic score
SREs: Skeletal-related events
